# Influence of simulated hypogravity on oxygen uptake during treadmill running

**DOI:** 10.14814/phy2.14787

**Published:** 2021-05-05

**Authors:** Kenan Yilmaz, Mark Burnley, Jonas Böcker, Klaus Müller, Andrew M. Jones, Jörn Rittweger

**Affiliations:** ^1^ Department of Muscle and Bone Metabolism Institute of Aerospace Medicine German Aerospace Center (DLR) Cologne Germany; ^2^ Endurance Research Group School of Sport and Exercise Sciences University of Kent Chatham Maritime United Kingdom; ^3^ Sport and Health Sciences College of Life and Environmental Sciences University of Exeter St Luke's Campus Exeter United Kingdom; ^4^ Department of Pediatrics and Adolescent, Medicine University of Cologne Cologne Germany

**Keywords:** countermeasures, microgravity, oxygen uptake, spaceflight, supine running, ${\dot{V}O}_{\text{2max}}$

## Abstract

Prolonged exposure to microgravity during spaceflights leads to severe deterioration in the physical performance of astronauts. To understand the effectiveness of existing in‐flight daily countermeasures and to plan exercise onboard the International Space Station, we compared supine treadmill running to traditional upright treadmill running on earth. Specifically, we assessed the cardiorespiratory responses to conventional upright running to the responses to supine treadmill running under 0.3 g, 0.6 g, and 1 g of body weight in younger (20–30 years, n = 14, 8 females) and older healthy adults (50–60 years, n = 12, 6 females). Maximal cardiorespiratory capacity was additionally evaluated by performing an incremental running protocol on each treadmill. Maximum speed was greater for 0.3 g and 0.6 g in supine than for upright running (18.5 km/h (1.1) and 15.9 (3.1) vs 13.2 (2.4) *p* < 0.001). In contrast, maximum oxygen uptake (${\dot{V}O}_{\text{2max}}$) and maximum heart rate (HR_max_) were greater in upright running than in all supine conditions (Upright treadmill running vs S1.0G vs S0.6G vs S0.3G, 41.7 ml kg^−1^ min^−1^ (7.2) vs 30.5 (6.6) vs 32.9 (7.0) vs 30.9 (5.2), *p* < 0.001 and 171 beats min^−1^ (14) vs 152 (24) vs 155 (20) vs 152 (18), *p* < 0.001, respectively). The reduction in ${\dot{V}O}_{\text{2max}}$ was remarkably similar across all three supine conditions, could not be increased by higher running speeds and can be well explained by reduced ground reaction forces (GRF). Thus, although a gravity‐related restriction of pulmonary gas exchange or perfusion of the legs when exercising in the supine position can be suspected, findings are also explicable on grounds of the vertical treadmill mechanics. Reduced loading will constitute a substantial limitation to ${\dot{V}O}_{2}$ in space with implications for crew health and the physical deterioration of astronauts.

## Graphical abstract

To better understand astronauts’ physical performance in reduced gravity, we studied heart rate and oxygen uptake responses in supine treadmill running under simulated microgravity conditions. The maximum achievable metabolic rate (${\dot{V}O}_{\text{2max}}$) was lower by 20% in supine running, as compared to upright running, and maximum heart rate (HRmax) was likewise reduced. Oxygen uptake (${\dot{V}O}_{2}$) values were well predicted by the level of gravity reduction and running speed. This is of relevance for astronaut health, as low metabolic rate during exercise compromises the expected health benefits for space travelers.

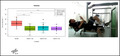

**New findings**

**What is the central question of this study?**

To better understand astronauts’ physical performance in reduced gravity, we studied heart rate and oxygen uptake responses in supine treadmill running under simulated microgravity conditions.

**What is the main finding and its importance?**

The maximum achievable metabolic rate (${\dot{V}O}_{\text{2max}}$) was lower by 20% in supine running, as compared to upright running and maximum heart rate (HR_max_) was likewise reduced. Oxygen uptake (${\dot{V}O}_{2}$) values were well predicted by the level of gravity reduction and running speed. This is of relevance for astronaut health, as low metabolic rate during exercise compromises the expected health benefits for space travelers.

## 1 INTRODUCTION

The human body has evolved in the permanent presence of Earth's gravity. Withdrawal of gravity during spaceflight results in a deconditioning process that can limit human presence in space (Baisden et al., 2008) and which can also pose serious threats to mission critical maneuvers, including re‐entry and landing (Michel et al., 1976). Especially during long‐term spaceflights over 5 month, a distinct cardiovascular deconditioning, consisting of reduced cardiac work and oxygen consumption, has been reported (Gallo et al., 2020). This deconditioning process is characterized by muscle wasting (Fitts et al., 2010), bone loss (Vico et al., 2000), reduced work capacity (Lawrence F. Dietlein (U.S.) and Igor D. Pestov (Russia), 2004) and orthostatic intolerance (Buckey et al., 1996; Kalinichenko, 1977; Wieling et al., 2002) with the latter likely resulting from adaptation of the cardiovascular system to decreased physical workloads and from the absence of a hydrostatic gradient (McArdle et al., 2015). To maintain their physical fitness and strength on board the International Space Station, astronauts engage in aerobic exercise on a treadmill and cycle ergometer and also participate in resistance exercise (Loehr et al., 2015; Seedhouse, 2020). For running exercise, they are strapped to the treadmill by a specific harness that distributes the force on the hips and shoulders (National Aeronautics and Space Administration (NASA), 2009; Novotny et al., 2013; National Aeronautics and Space Administration (NASA), 2019).

The typical pull‐down force, that is applied by means of bungees from the harness towards the treadmill's surface on board the International Space Station, is substantially lower than the astronauts’ weight on Earth (Genc et al., 2010; Gosseye et al., 2009). Thus, musculoskeletal forces, which constitute an important determinant for the maintenance of muscle, bone, and tendon (Rittweger, 2019), must be expected to be reduced in space. This could well explain why the current countermeasure exercises fail to prevent muscle atrophy and bone loss (Gruber et al., 2019; Korth, 2015; Rittweger et al., 2018). In addition, reduced pull‐down force naturally diminishes the external mechanical work performed during locomotion (Pavei et al., 2015), which would be expected to reduce the cardiorespiratory demands for a given speed of locomotion. This is important, as it would minimize the health benefits normally associated with running (Hespanhol Junior et al., 2015; Myers et al., 2004). However, it is still unknown to what extent the cardiorespiratory responses to running in microgravity might be blunted by simulated earth‐like g‐loading compared with usual treadmill running on earth. Finally, the exact quantitative relationship between the level of gravity reduction and cost of locomotion is not well established and it has never been studied on a verticalized treadmill.

The European Space Agency has devised a system that combines a supine treadmill facility with a subject loading system (SLS) that uses pressurized cylinders to generate an adjustable constant pull‐down force (Gosseye et al., 2010). Participants are strapped towards the running surface via a shoulder‐hip harness so that they can run with their body suspended in supine position. Importantly, there is no hydrostatic pressure gradient along the body's z‐axis when running in a supine position and pull‐down forces can be set to values typical of the International Space Station treadmill. In addition to being an elegant ground‐based model for International Space Station countermeasure exercise, the supine treadmill facility also offers the opportunity for hypogravity simulations on Earth to elucidate the possible physiological responses to sojourns to the Moon or Mars (Weber et al., 2019).

We were therefore interested to assess the maximal and submaximal cardiorespiratory responses (Hawkins et al., 2007) to treadmill running in the supine body position with the application of various pull‐down forces. Given the well‐established reduction in external work during hypogravity, we hypothesized that HR_max_ and ${\dot{V}O}_{\text{2max}}$ would be lower than upright treadmill running on a standard treadmill (experimental question 1). We also hypothesized that the HR and ${\dot{V}O}_{2}$ responses to running would be affected by the pull‐down force (experimental question 2), given the importance of external biomechanical work for the cost of human locomotion. To obtain results that represent a wider population and possible age‐related differences, we investigated these questions in a mixed cohort of younger and older healthy adults since the mean age of astronauts is between 42.5 and 44.5 years (Goel et al., 2014) and returning astronauts from longer space mission are dealing with health problems that are also common in elderly (Strollo et al., 2018).

## 2 MATERIALS AND METHODS

### 2.1 Ethical approval

This study was approved by the ethical committee of the Medical Council North‐Rhine (Ärztekammer Nordrhein / reference number: 2013350) in Düsseldorf, Germany. Written informed consent was given by the participants before being included into the study that conformed to the standards set by the latest revision of the Declaration of Helsinki, except registration in a database, at the time of the study.

The study was conducted in the Physiology laboratory of the Institute of Aerospace Medicine in the department of space physiology at the German Aerospace Center (DLR) in Cologne, Germany.

### 2.2 Participants

Twenty‐six recreationally active participants were recruited and placed into one of four groups, namely: younger women, older women, younger men and older men. Before study admission, participants received a standard medical screening and a ${\dot{V}O}_{\text{2max}}$ test was performed using the “Bruce” protocol (Pollock et al., 1976) to ascertain their aerobic fitness. Inclusion criteria were as follows: age between 20 to 30 years (younger women and younger men) or 50 to 60 years (older women and older men) to evaluate possible age‐related differences in responses; body mass index (BMI) in the range of possible astronauts between 18 kg/m^2^ and 28 kg/m^2^ (National Aeronautics and Space Administration (NASA), 1995); ${\dot{V}O}_{\text{2max}}$ equal to or greater than 30 ml kg^−1^ min^−1^ for younger women, 25 ml kg^−1^ min^−1^ for older women, 40 ml kg^−1^ min^−1^ for younger men and 35 ml kg^−1^ min^−1^ for older men, so that a comparison of physiological responses at higher speeds was also possible. Key exclusion criteria were drug or alcohol abuse, intake of any medication during the study, smoking, and being a competitive athlete.

For estimation of the sample size, a ${\dot{V}O}_{\text{2max}}$ reduction by 5% was considered as meaningful. Thus, a sample size estimation was performed with the R‐package “pwr” (www.r‐project.org) and we used previously published data on means and SD of ${\dot{V}O}_{\text{2max}}$ (Wilkerson et al., 2005). Setting α to 0.05 and β to 0.2, we obtained a sample size of 24. In order to accommodate for drop‐out, a total of 28 subjects were enrolled. Although the study was not powered to detect effects of age or gender, we recruited younger and older men and women in order to be inclusive of a wider age spectrum and of both sexes.

### 2.3 Study conduct

The running protocols were conducted between January 2014 and March 2017 and required each participant to visit the laboratory on five occasions (supporting information: Table 4). The first visit (V0) assessed ${\dot{V}O}_{\text{2max}}$ by the Bruce protocol and familiarized participants with running on the supine treadmill facility. The second visit (V1) involved participants completing an incremental test with the speed profile shown in (supporting information: Table 5) on a standard treadmill (Schiller MTM‐1500 med, manufactured by H/P/COSMOS, Nussdorf‐Traunstein, Germany).

**FIGURE 1:**
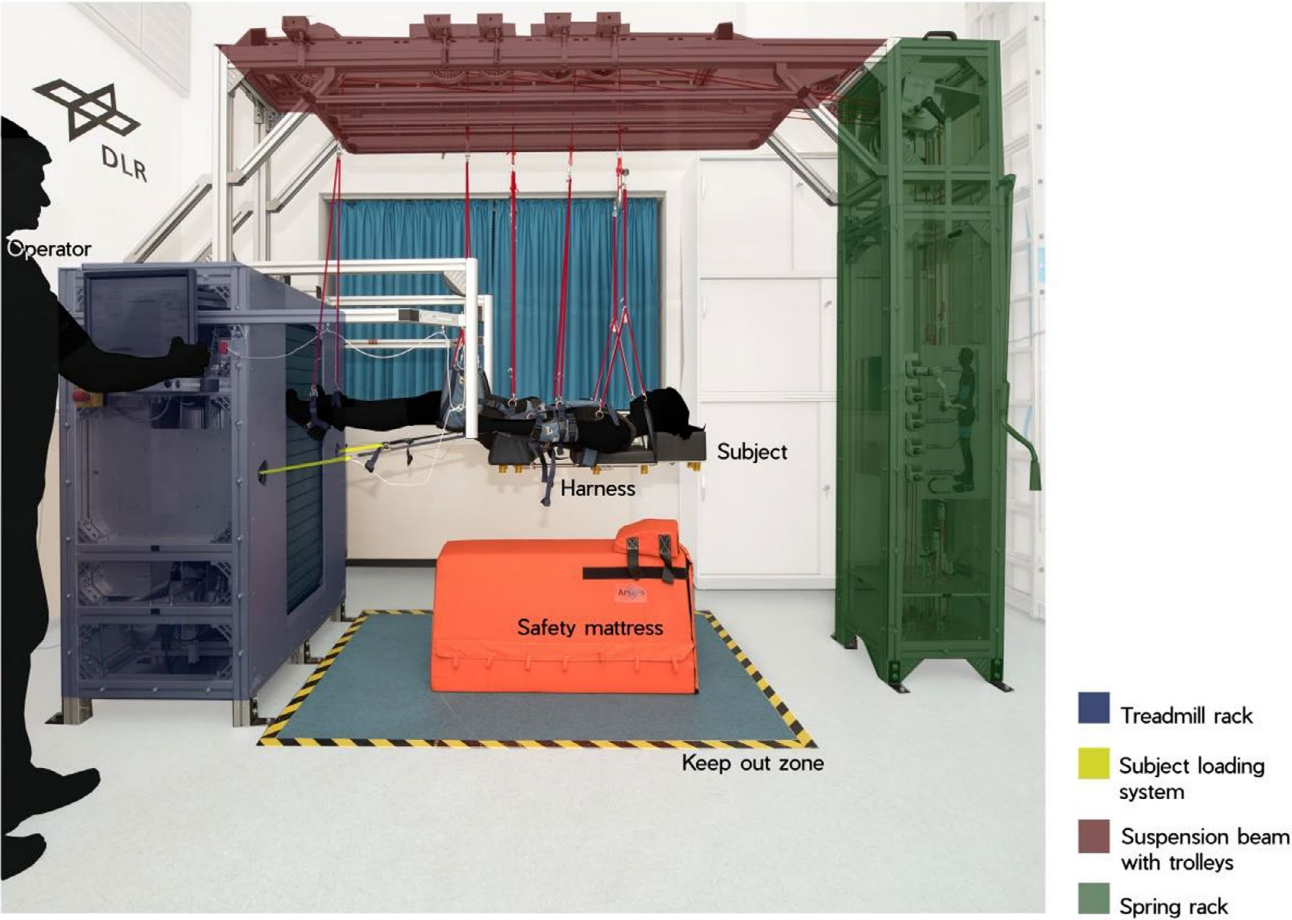
Supine treadmill facility: Overview of the supine treadmill facility and subject loading system (SLS): A customized, commercially available “Woodway”‐treadmill is mounted vertically into a chassis. The suspension system is mounted to the chassis and hosting the harness to negate the effects of Earth's gravity on the participant. The SLS provides a constant pull down force on the locomotion surface with the help of pneumatic cylinders and ropes

This speed profile consisted of an initial phase for assessing the ${\dot{V}O}_{2}$ kinetics that started with 5 minutes of rest and was followed by 5 minute steps at speeds of 25% and 50% of the ${\dot{V}O}_{\text{2max}}$ that was previously determined using the Bruce protocol at V0. This was followed after 5 minutes of rest by an incremental phase. In this phase, the running speed was increased every 4 minutes by 2.5 km/h starting at a speed of 4 km/h and rising to a maximum of 19 km/h. The remaining visits (V2‐V4) were performed on the supine treadmill facility (Figure 1 and subsection “supine treadmill facility”) with the same speed profile as during V1, but with three different levels of pull‐down force, equating to 0.3 g, 0.6 g, and 1 g. Notably, the sequence of pull‐down forces administered was randomized within participants in order to balance any learning effects. To randomize the three different pull‐down forces, closed envelopes with all six possible different running sequences (3 + 2+1) were prepared and drawn for each participant. The mean time period the participants completed V1‐V4 was 32.5 days (29.5). For all running tests in V0‐V4, the criteria for termination were one of the following: achievement of predicted HR_max_ (220 ‐ age), ${\dot{V}O}_{2}$ plateau, angina pectoris, ischemic signs in the ECG, arrhythmias (complex extrasystoles, atrioventricular block II° or III°), signs of circulatory disturbance (cyanosis, paleness), respiratory insufficiency, dizziness, exhaustion or upon request of the participant.

### 2.4 Supine treadmill facility

The supine treadmill facility has been described in detail elsewhere (Gosseye et al., 2010). In brief, the facility (Figure 1) consists of three components, namely: a) a second generation treadmill (T2) as used on the International Space Station (Penta, 2008), mounted vertically into a chassis to provide a locomotion surface; b) a suspension system, mounted to the chassis and hosting the harness to negate the effects of Earth's gravity on the participant; and c) the SLS that provides a constant pull‐down force on the treadmill surface with the help of pneumatic cylinders and ropes. The SLS generates the pull‐down force by means of two pneumatic cylinders, and the force variation of the pistons is ±5%. Two ropes were connected to the harness that loaded the shoulder and the hip in a 1/3 to 2/3 ratio, and the rope tension was set via the in‐built software to 30%, 60%, or 100% body weight, that is, 0.3 g, 0.6 g, and 1.0 g.

### 2.5 Endpoint measurements

Running speed and pull‐down force data were continuously generated by the software integrated into the supine treadmill facility. ${\dot{V}O}_{2}$was assessed with a MetaLyzer (Cortex Biophysik Leipzig GmbH) under stable environmental conditions and the integrated software MetaSoft in its version 3.9.7 SR 4. This is a stationary spiroergometry system that calculates respiratory volume, O_2_ uptake and CO_2_ output breath‐by‐breath. The system was calibrated according to the manufacturer's instructions. An ambient air measurement was made before each test. The volume transducer was calibrated, a one point gas analyzer calibration was carried out before each test and a two point gas analyzer calibration was done every other day. ECG was recorded via a 12 channel ECG (cardio 100 BT, custo med, Ottobrunn, Germany). Blood lactate was measured in capillary samples taken from the ear lobe before and during the protocol at 3 min, 6 min, and 9 min after the end of the cool down phase. The ECG was synchronized to the MetaSoft data via the MetaSoft software and synchronization of the vertical treadmill data with the MetaSoft data was achieved through setting the system clock of the two computers.

### 2.6 Data processing and statistical analysis

Data processing and statistical analyses were performed with the Software “R” (http://www.r‐project.org), version 3.5.1. All data were visually inspected prior to analyses. A factor “condition” was generated to reflect running during either supine position (S0.3 g, S0.6 g, S1.0 g for running with a pull‐down force equivalent to 0.3 g, 0.6 g, and 1.0 g, respectively) or during upright treadmill running. Pull‐down forces and GRF were averaged epoch‐wise for each condition and participant. Division of ${\dot{V}O}_{2}$ by HR yielded the oxygen pulse (thus ml of ${\dot{V}O}_{2}$ per heart beat), and division of CO_2_ output (${\dot{V}\text{CO}}_{2}$) by ${\dot{V}O}_{2}$ yielded the respiratory exchange ratio (RER). Cardiorespiratory endpoints were assessed in two ways. Firstly, for all 3‐minute increments that had been completed during the testing, HR, ${\dot{V}O}_{2}$, ${\dot{V}\text{CO}}_{2}$, RER, and oxygen pulse for a given condition and speed were extracted as the arithmetic mean during the final 60 s of the increment. These data will be referred to as increment‐data. In order to achieve peak data for each condition, data were averaged over the last 60 seconds before termination of the last individual increment. Blood lactate was calculated by the mean of the peak values per participant and condition.

The ${\dot{V}O}_{2}$ kinetics analysis was performed using Graphpad Prism 8. Breath‐by‐breath data were manually edited to remove errant breaths due to coughing or swallowing (defined as a value 4 SD greater/less than the mean of the preceding 5 breaths). After removal of the first 20 s of data to avoid contamination by the cardiodynamic component of the ${\dot{V}O}_{2}$ kinetics, a mono‐exponential function was applied to the on‐transient of the form:

(1) $${\dot{V}O}_{2}\left(t\right)={\dot{V}O}_{2}\left(b\right)+A_{P}*\left(1‐e^{‐\left(t‐\text{TD}\right)/\tau }\right)$$

where ${\dot{V}O}_{2}\left(t\right)$ is the ${\dot{V}O}_{2}$ at time t, ${\dot{V}O}_{2}\left(b\right)$ is the baseline ${\dot{V}O}_{2}$ (defined as the mean of the final minute of baseline (rest) in the first transition). The A_P_ parameter is the primary amplitude above baseline, TD the time delay before the onset of the exponential function and τ is the time constant of the response. The amplitude of the response to the second transition was too small to provide meaningful kinetic data in most cases, and therefore, the ${\dot{V}O}_{2}$ kinetics was assessed using the first transition only.

For statistical analyses of cardiopulmonary and demographic data during ${\dot{V}O}_{\text{2max}}$ testing, linear mixed‐effect models were used (function “lme” from the R‐library “nlme”) with participant as random factor and age group and sex as fixed factors (Table 1), including the interaction term. For model simplification, we first eliminated the interaction term and then the main term with greatest *P*‐value as long as the respective *p*‐value was >0.2. Significant findings were followed up by treatment *a priori* contrasts, using S1.0 g as reference. In order to compare cardiorespiratory peak data across gravitational condition (S0.3 g, S0.6 g, S1.0 g or Upright treadmill running), we also used the lme function with participant as random, and condition and group as fixed effects, again including a term for their interaction. Here, model simplification eliminated the interaction term first and the group term next. Again, follow‐up of significant effects was performed with treatment contrasts with S1.0 g as reference.

**TABLE 1:** Demographic characteristics and cardiorespiratory responses to ${\dot{V}O}_{\text{2max}}$ testing in Upright treadmill running with the Brue protocol; “n”** = **number of participants

	Older Women	Older Men	Younger Women	Younger Men
Age^AAA,G^ [Years]	52.2 (1.5) (n = 6)	54.5 (1.2) (n = 6)	23.4 (2.3) (n = 8)	25.2 (2.3) (n = 6)
Height^GGG^ [cm]	165.5 (5.2) (n = 6)	176.8 (3.9) (n = 6)	165.6 (3.7) (n = 8)	180.5 (5.2) (n = 6)
Weight^GGG^ [kg]	57.8 (2.3) (n = 6)	76.5 (8.2) (n = 6)	59.9 (5.6) (n = 8)	76.5 (11.6) (n = 6)
BMI^GG^ [kg/m^2^]	21.0 (1.1) (n = 6)	24.5 (2.4) (n = 6)	21.8 (1.8) (n = 8)	23.5 (3.0) (n = 6)
Speed^A^ [km/h]	7.1 (0.7) (n = 6)	6.9 (0.5) (n = 6)	7.6 (0.9) (n = 7)	7.9 (0.7) (n = 6)
${\dot{V}O}_{2}$ max ^GGG^ [ml min^−1^ kg^−1^]	39.9 (5.5) (n = 5)	43.3 (7.7) (n = 6)	43.9 (6.3) (n = 8)	50.1 (8.8) (n = 6)
HR_max_^A^ [min^−1^]	169 (12) (n = 6)	166 (7) (n = 6)	177 (14) (n = 8)	183 (10) (n = 6)
RER	1.20 (0.09) (n = 5)	1.13 (0.08) (n = 6)	1.21 (0.05) (n = 8)	1.20 (0.09) (n = 6)
O_2_‐Pulse^GGG^ [ml O2 heartbeat^−1^]	13.2 (1.3) (n = 5)	19.8 (1.8) (n = 6)	15.0 (2.8) (n = 8)	21.0 (4.7) (n = 6)

Note

Data are given as means and standard deviation (in brackets). ^AAA^, ^AA^, and ^A^ denote significant effects of age with *p* < 0.001, *p* < 0.01, and *p* < 0.05, respectively. ^GGG^, ^GG^, and ^G^ denote significant effects of sex with *p* < 0.001, *p* < 0.01, and *p* < 0.05, respectively.

Data are given as means and their SD, if not indicated otherwise, and the level of statistical significance was set to *p* < 0.05.

## 3 RESULTS

### 3.1 Participants’ characteristics and peak cardiorespiratory responses during ${\dot{V}O}_{\text{2max}}$ testing in standard upright treadmill running

Twenty‐six volunteers participated in this study, with six older women, six older men, eight younger women and six younger men. Their anthropometric characteristics are given in Table 1. All participants had to run under the four different conditions. Fourteen (13%) out of the planned 104 runs were not completed due to participant drop‐out because of the timetable and unsafe running feeling (on the usual treadmill) or due to technical problems during the testing sessions leading to a total of 90 runs.

Analysis of anthropometric data revealed the expected effects of sex in body height and BMI (*p* < 0.001 and *p* < 0.01, respectively). Statistical analysis of the Bruce protocol data showed that older groups had lower values for Speed (*p* < 0.05) and HR_max_ (*p* < 0.05) but failed to reveal an age effect for ${\dot{V}O}_{\text{2max}}$ (*p* = 0.281), RER (*p* = 0.185) and oxygen pulse (*p* = 0.657). Moreover, women had a lower ${\dot{V}O}_{\text{2max}}$ (*p* < 0.001) and oxygen pulse than men (*p* < 0.001).

### 3.2 Peak cardiorespiratory responses in relation to gravitational condition

**FIGURE 2:**
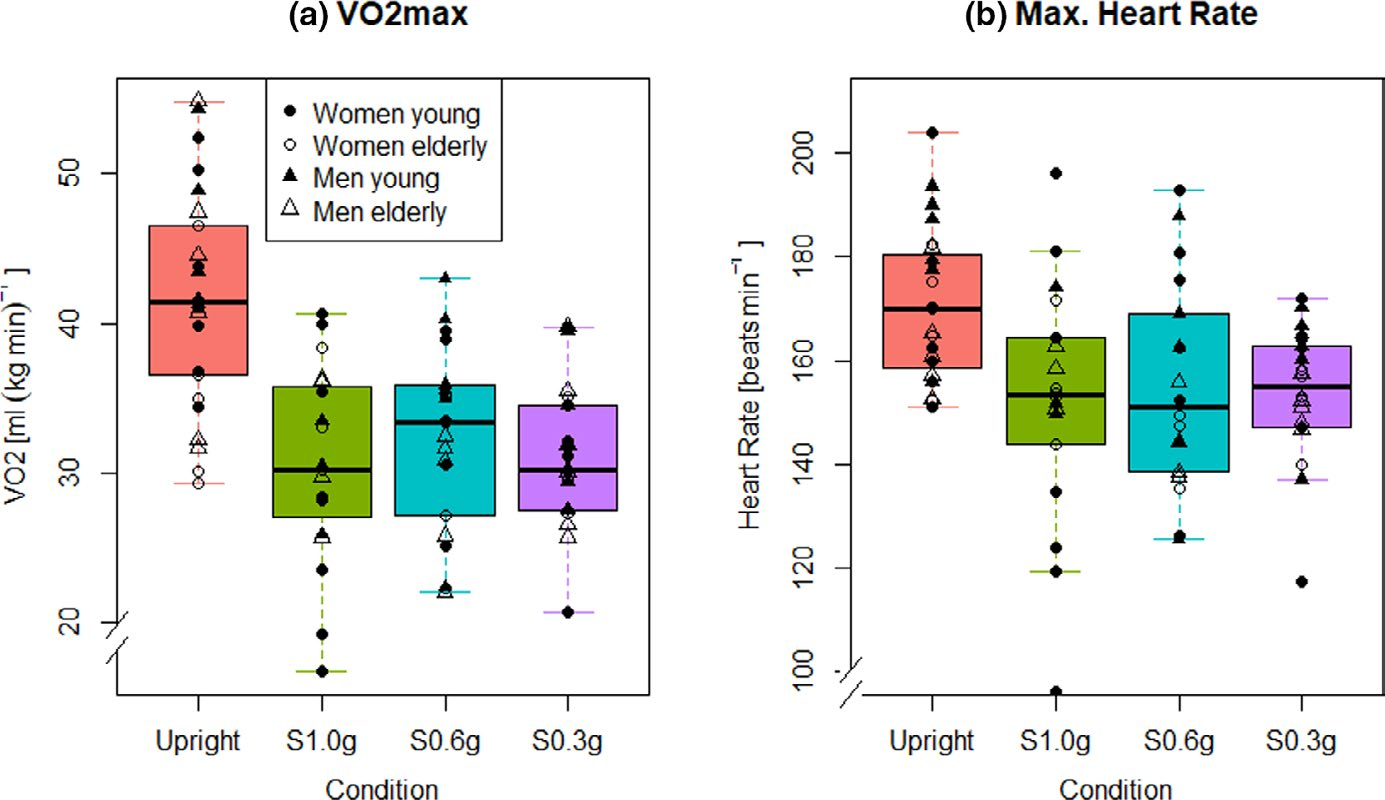
${\dot{V}O}_{\text{2max}}$Cardiopulmonary responses in relation to the four different gravitational conditions. a) Oxygen uptake and b) Maximal heart rate. “Upright” = Upright treadmill running, “S1.0 g”; “S0.6 g”;“S0.3 g” = supine running under a load of 1 g; 0.6 g, and 0.3 g of body weight. Whisker plot reference: solid line = median (50 percentile), upper line = quartile (25 percentile), lower line = quartile (75 percentile), line between lower and upper quartile = interquartile range, disconnected lines = upper and lower outlier range

Statistical analyses of the peak running protocol data showed that ${\dot{V}O}_{\text{2max}}$ was reduced in the supine running conditions as compared to upright treadmill running (*p* < 0.001), without any difference among the three supine conditions (*p* > 0.20, see Table 2 and Figure 2). Likewise, HR_max_ (*p* < 0.001), RER (*p* < 0.001) and oxygen pulse (*p* < 0.001) were all greater during upright treadmill running than during supine treadmill running, without any difference across the three supine conditions (all *p* > 0.40). No effects of age or sex were found for ${\dot{V}O}_{\text{2max}}$, HR_max_ or RER, but women had lower oxygen pulse than men (*p* < 0.001) across age groups. Maximum running speed differed across the different gravitational conditions (*p* < 0.001), with values being lower for supine treadmill running at 1 g than for upright treadmill running (*p* < 0.05) and also lower than for the two supine hypo‐g conditions (both *p* < 0.001, see Table 2 and supporting information: Figure 4).

**TABLE 2:** Cardiorespiratory responses to treadmill running under different gravitational conditions

	Upright	S1.0 g	S0.6 g	S0.3 g
Speed_max_^***^ [km/h]	13.2^a^ (2.4) (n = 26)	11.5 (2.6) (n = 19)	15.9^aaa^ (3.1) (n = 22)	18.5^aaa^ (1.1) (n = 23)
${\dot{V}O}_{\text{2max}}$ ^***^ [ml kg^−1 ^min^−1^]	41.7^aaa^ (7.2) (n = 26)	30.5 (6.6) (n = 19)	32.9 (7.0) (n = 22)	30.9 (5.2) (n = 23)
Heart Rate_max_^**^ [min^−1^]	171^aa^ (14) (n = 24)	152 (24) (n = 18)	155 (20) (n = 18)	152 (18) (n = 22)
RER^*^ [${\dot{V}\text{CO}}_{2}$/ ${\dot{V}O}_{2}$]	1.06^aa^ (0.08) (n = 26)	0.98 (0.1) (n = 19)	0.98 (0.12) (n = 22)	0.99 (0.09) (n = 23)
O_2_‐Pulse^***^ [ml O_2_ /heartbeat]	16.4^aa^ (3.5) (n = 24)	12.7 (2.4) (n = 18)	14.3 (2.9) (n = 18)	13.7 (2.7) (n = 22)
Blood Lactate^*^ [mmol l^−1^]	4.36^a^ (2.21) (n = 24)	3.23 (1.60) (n = 23)	3.22 (1.82) (n = 22)	2.89 (1.67) (n = 23)
G‐load from pull‐down force^***^ [g]	1 (set to 1)	1.02 (0.1) (n = 168 episodes)	0.62^aaa^ (0.0) (n = 274 episodes)	0.31^aaa^ (0.0) (n = 251 episodes)
G‐load from ground reaction force^***^ [g]	1^aaa^ (set to 1)	0.89 (0.0) (n = 88 episodes)	0.54^aaa^ (0.0) (n = 169 episodes)	0.25^aaa^ (0.1) (n = 158 episodes)

Note

“n” = number of participants, “Speed_max_” = Maximum speed in [km/h], “${\dot{V}O}_{\text{2max}}$” = maximum oxygen uptake in [ml kg^−1 ^min^−1^], “Heart Rate_max_” = maximum heart rate [min^−1^], “RER” = respiratory exchange ratio in [${\dot{V}\text{CO}}_{2}$/ ${\dot{V}O}_{2}$], “Oxygenpulse” = “O_2_‐Pulse” in [ml O_2_/ heartbeat], Blood lactate in [mmol l^−1^], measured during running upright and during supine running under a g load of “S1.0 g” =1.0 g of body weight, “S0.6 g” =0.6 and “S0.3 g” =0.3 g of body weight. “*”, “**”, “***” denote significant main effect for condition with *p* < 0.05, *p* < 0.01 and *p* < 0.001, respectively. “a”, “aa”, “aaa” denote significant different from S1.0 g condition with *p* < 0.05, *p* < 0.01, and *p* < 0.001, respectively. No significant condition*group interaction terms were found (all *p* ≥ 0.20).

When verifying the pull‐down force levels for the different supine running conditions, it was found that the averaged pull‐down force exceeded the target force by a small amount, namely by 1.3% (SD 2.1%, *p* < 0.001). By contrast, the averaged GRF during running was 20.9% (SD 35.3%) below the target pull‐down force (*p* < 0.001), which was an unexpectedly large difference (see Table 3). This suggests that substantial hysteresis occurred within the pneumatic actuator of the SLS that made test participants “float” during supine treadmill running. Hence, for the further analysis of the incremental data, we focused on both running speed and GRF as independent variables.

**TABLE 3:** Metabolic cost of running in (J·kg^−1^·m^−1^) in the different gravitational conditions, expressed as mean values (SD)

	Treadmill speed [km/h]					
	4	6.5	9	11.5	14	16.5
Upright	2.11 (0.41) (n = 26)	3.23 (0.59) (n = 26)	3.53 (0.38) (n = 26)	3.55 (0.36) (n = 23)	3.4 (0.27) (n = 16)	3.51 (0.15) (n = 4)
S1.0 g	2.26 (0.45) (n = 19)	2.69 (0.81) (n = 19)	2.78 (0.56) (n = 19)	2.71 (0.47) (n = 12)	2.61 (0.4) (n = 7)	
S0.6 g	1.6 (0.67) (n = 22)	2.02 (0.53) (n = 22)	2.04 (0.54) (n = 22)	1.92 (0.51) (n = 21)	1.91 (0.48) (n = 19)	2.03 (0.47) (n = 15)
S0.3 g	1.26 (0.29) (n = 23)	1.28 (0.24) (n = 23)	1.31 (0.24) (n = 23)	1.35 (0.24) (n = 23)	1.48 (0.24) (n = 23)	1.68 (0.34) (n = 23)

Note

No value is given for 16.5 km/hour at S1.0 g, as only one participant mastered this stage. ANOVA demonstrated effects of speed, condition, and their interaction (all *p* < 0.001), and a priori contrasts demonstrated that the condition effect for S1.0 g differed from S0.6 g and S0.3 (both *p* < 0.001), while the interaction effect differed between S1.0 g and Upright treadmill running (*p* < 0.001).

**FIGURE 3:**
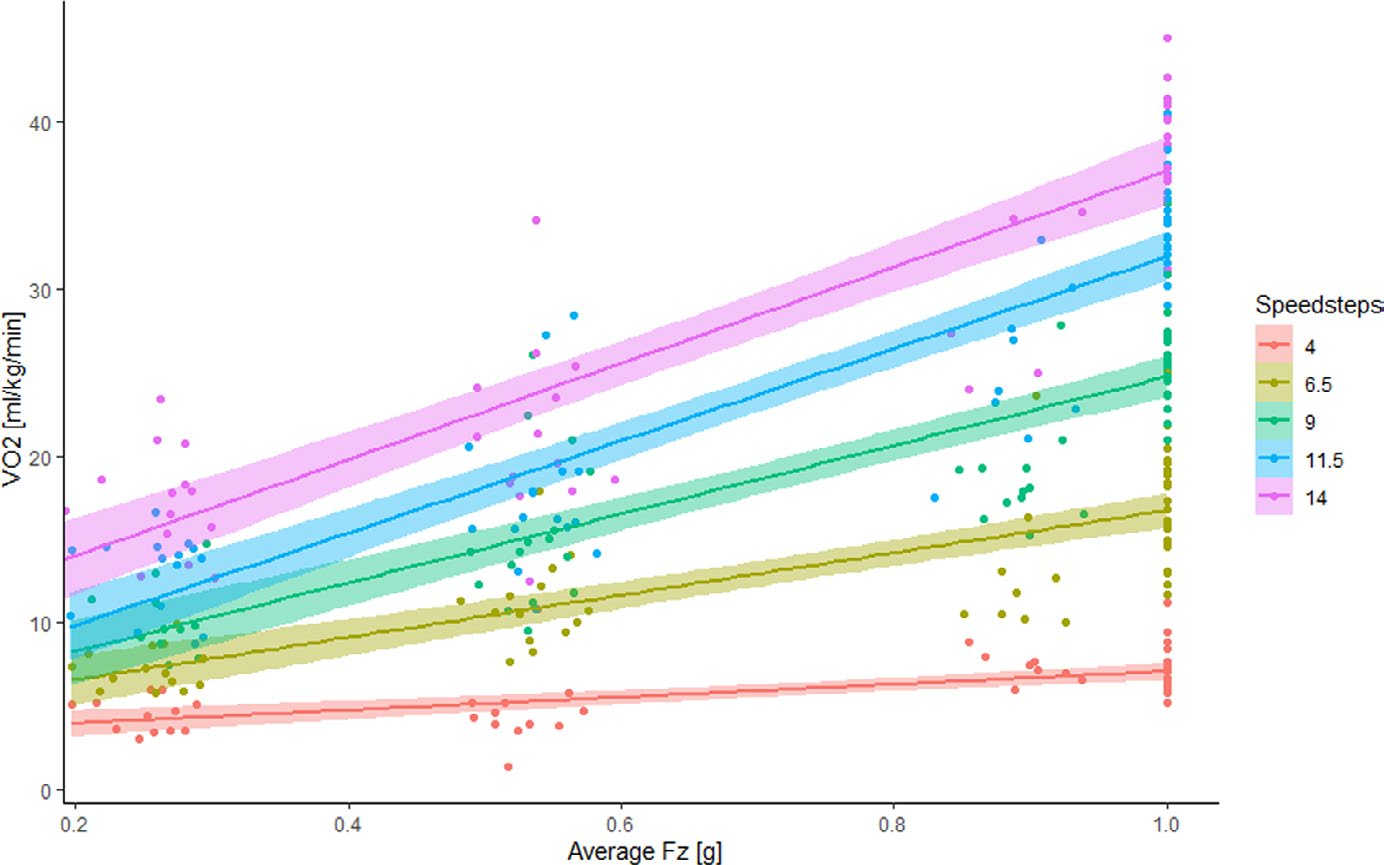
Net oxygen uptake (${\dot{V}O}_{2}$) plotted against the averaged ground reaction force (GRF), normalized to body mass and given in g. Data are separately displayed for all running speed‐steps from 4 to 14 km/h. Note that GRF was set =1 for upright running, but measured for supine running. The solid lines denote the regression lines of each speed level whilst the shaded colors represent the standard errors with 95% of confidence interval

Figure 3 therefore displays ${\dot{V}O}_{2}$ values for each running speed, plotted as a function of GRF. When fitting a linear mixed‐effect model with ${\dot{V}O}_{2}$ as dependent and speed and GRF (normalized to body mass) as independent variables, residuals for upright treadmill running were not significantly different from residuals from the three supine running conditions (*p* = 0.62), suggesting that reduced GRFs can fully explain the reduced ${\dot{V}O}_{2}$ values in supine conditions. The mixed‐effect model yielded beta‐coefficients −4.00 (SE 1.22), 5.57 (1.95), 1.18 (SE 0.08), and 1.00 (SE 0.17) for intercept, reaction force, speed, and the force*speed interaction term, respectively.

The phase II ${\dot{V}O}_{2}$ kinetics time constant, τ, was 29.6 (15.6) s, 19.5 (9.4) s, 22.4 (10.4) s, and 38.5 (22.1) s for upright treadmill running, S1.0 g, S0.6 g, and S0.3 g, respectively. ANOVA revealed that these values were different (*p* < 0.001), and *a priori* contrast demonstrated differences between S1.0 g and upright treadmill running (*p* = 0.024), and also between S1.0 g and S0.3 g (*p* < 0.001).

## 4 DISCUSSION

Our primary aim was to determine whether supine running with various pull‐down forces (i.e., in different conditions of simulated microgravity) produces different maximal cardiorespiratory responses (${\dot{V}O}_{\text{2max}}$ and HR_max_) compared with regular treadmill running in an upright position. The first finding of the present study is that throughout all supine conditions, ${\dot{V}O}_{\text{2max}}$ and HR_max_ did not reach the same level as for upright treadmill running (Hypothesis 1). Indeed, running in supine position was associated with a lowering of the maximally achievable metabolic rate by more than 20% (Tables 2 and 3). Additionally, and only when loading attempted to achieve full body weight (simulating 1.0 g), a reduction in maximum running speed was a secondary contributor to blunted peak metabolic rate in the supine position. This is of relevance for astronaut health, as lowering of metabolic rate during aerobic exercise would be expected to compromise the expected health benefits of treadmill exercise.

The second finding of our study was that the exercise‐related ${\dot{V}O}_{2}$ can be accurately modeled as a function of running speed and GRF (Figure 3). While previous studies had suggested that the walk‐run transition and the optimal speed scale with the square root of gravity (Minetti, 2001), the relationship between pull‐down force and metabolic demand seems quite linear in the present data. In any case, it seems that the lowered ${\dot{V}O}_{\text{2max}}$ in supine treadmill running is well explained by the reduction in GRF during supine running at 1.0 g as compared with upright running.

### 4.1 Are physiological responses altered in supine exercise?

Since the vertical treadmill facility with the subject loading system for ground‐based testing has been recently invented and in looking from an historical perspective, it seems well established for cycling exercise that ${\dot{V}O}_{\text{2max}}$ is reduced in the supine position, as compared to the sitting position. Several studies (Astrand & Saltin, 1961; Proctor et al., 1996; Takahashi et al., 1998) report reductions which range from 8% up to 29% between upright and supine maximal cycling exercise.

Concerning the supine running condition, a possible reduction in postural muscle recruitment can be discussed. But we have to be aware to postulate the postural muscles as the ones that maintain erect standing on one leg against gravity during walking or running for most time of the gaitcycle, since it is known that for just standing well balanced on two legs, almost no muscle activity is needed except against gravity (Goss, 1963; Janda, 1983). Consequently possible lower postural muscle recruitment would correspond to the grade of gravity reduction in pull‐down force. According to this, it has been recently found that trunk stiffness was reduced in hypogravity, with weaker compensating paraspinal muscles and higher activation of the deep abdominal muscle (De Martino et al., 2020).

The impediment to ${\dot{V}O}_{\text{2max}}$ in the supine position is interesting for two reasons. Firstly, venous return is obviously facilitated (Takahashi et al., 2005). Secondly, an improvement in ${\dot{V}\text{CO}}_{2}$ dissipation relative to minute ventilation has been observed and interpreted as a sign of reduced dead space ventilation, or as an indicator of improved diffusion capacity of the lung in the supine position (Nakamura et al., 1998). Admittedly, these effects seem to be more pronounced at lower metabolic rates, and they seem to level off toward the maximal rate (Leyk et al., 1994). However, a lower perfusion pressure gradient occurs as a consequence of the lower hydrostatic pressure during exercise in the supine posture, which leads to a reduction of net driving pressure in the extremities (Laughlin & Joyner, 2003; Leyk et al., 1994; Rowell, 1974). As a consequence, compared with supine cycling, the arterial blood flow in the legs in upright cycling is higher (Shiotani et al., 2002).

Although in contradiction to the general assumption of impeded ${\dot{V}O}_{\text{2max}}$ in supine cycling, Leyk et al. (1994) argue that many of the previous studies had not been careful enough in matching the biomechanical aspects of cycling in the supine and sitting positions. Thus, when creating a neat biomechanical match by tilting the same ergometer between conditions, the authors found only a small (−5%) decrement, which was non‐significant with n = 9. Our study confirms this interpretation, and one would predict from our statistical analyses that ${\dot{V}O}_{2}$ could be comparable during upright and supine running, if it were possible to achieve the same GRFs in either condition.

### 4.2 Using a verticalized treadmill to study hypo‐gravity locomotion

Given the relevance of external work for the metabolic cost of running (Cavagna et al., 1964), it seems logical that the cost of running was reduced, as it is a function of GRF. As a consequence, reductions in the cost of locomotion with reduced gravity have been repeatedly described in various previous studies that have simulated reduced gravity conditions on Earth (Farley & McMahon, 1992b; Griffin et al., 1999; Pavei et al., 2015; Ueberschär et al., 2019). However, these previous studies tested partially suspended human participants in the upright position. The question therefore arises whether upright and supine models of hypogravity locomotion yield comparable results.

With their suspension hypogravity model, Farley & McMahon (1992a) compared walking (1 m/s) and running (3 m/s) at 0.25, 0.5, 0.75, and 1 g. It was reported that ${\dot{V}O}_{2}$’s gravity dependency was greater for running than for walking. The present study expands this and demonstrates a continuous scaling of ${\dot{V}O}_{2}$ with gravity and locomotion speed, including running as well as walking.

### 4.3 ${\dot{V}O}_{2}$ kinetics

In reference to the ${\dot{V}O}_{2}$ kinetics, during the transient part of the running protocol rising to 25% of ${\dot{V}O}_{\text{2max}}$, we observed that τ was faster in S1.0 g and S0.6 g than in the upright position. This is in contrast to former studies, in which a reduction in local blood flow in the supine position causes a slower increase in ${\dot{V}O}_{2}$ (MacDonald et al., 1998). It is important to note that the ${\dot{V}O}_{2}$ kinetic data were estimated from single transitions to a relatively low fraction of the ${\dot{V}O}_{\text{2max}}$, and thus, the confidence limits were rather wide (mean ± SD 95% CI, 9 ± 9 s). Nevertheless, the amplitude of these responses was consistent with the lower ${\dot{V}O}_{2}$ values reported during the incremental test when microgravity was simulated.

The phase II time constant is believed to be predominantly related to an intrinsic metabolic inertia but can be slowed further in situations where muscle O_2_ supply may be limited, thus increasing the phase II τ (Poole and Jones, 2012). Accordingly, slower ${\dot{V}O}_{2}$ kinetics in the supine running conditions were expected due to the likely impairment of perfusion consequent to body position (Denis & Perrey, 2006). Surprisingly, however, we found longer time constants in upright as compared to horizontal running at 1 g, and a slowing of the uptake kinetics was observed when reducing the pull‐down force from 1 g to 0.3 g in supine running.

A possible explanation for the shorter time constant in the S1.0 g compared with upright treadmill running could be the activation of different muscle fiber types (Linnarsson, 2009) which are known to play an important role in the efficiency of energy utilization (Moritani et al., 1993; 2009). For example, a higher activation of type I muscle fibers is related to faster ${\dot{V}O}_{2}$ kinetics (Barstow et al., 1996). A possible greater use of the upper body could also occur in the supine conditions to prevent the body from rotating around the z‐axis especially at lower speeds and higher g load. The rotation around the z‐axis stabilizes at higher running speeds. This leads to possible inefficient running at lower speeds in the supine condition with a greater metabolic demand. If this was to occur early in exercise before resolving itself later, this would stimulate an apparently faster rise initial in ${\dot{V}O}_{2}$. Overall, the ${\dot{V}O}_{2}$ kinetics results are surprising but are likely explained by methodological limitations as well as differences in metabolic demand, body position and running technique between conditions.

### 4.4 Implications for the International Space Station

In the present study, it would have been impossible to further increase the pull‐down force, because test participants were already quite strained by the harsh shoulder pressure in the S1.0 g condition. It is clear to us that this limitation will also occur when loading harnesses are used in space and that it will generally be very difficult to apply forces that are greater than 1 g through interfaces other than the feet. This results in low GRF and foot forces during treadmill running on the International Space Station (Genc et al., 2006) (Cavanagh et al., 2010). Another limitation consists of the ground‐based testing on earth itself, because these facilities can only simulate and not replicate fully the microgravitational environment.

Regarding the GRF, it is likely that the reduction in maximal metabolic rate is associated with GRF in the SLS. In our case, it is important that the external loading by the SLS was not reduced. To be sure of the GRF to be the cause of the lower metabolic rate, another study for a control experiment could be either to measure also GRF in upright treadmill running or to raise the loading in the SLS over 100% of body weight till the GRF between upright treadmill running and supine treadmill running are equal and measure the cardiorespiratory responses again. Alternatively, upright treadmill running with a certain degree of weight support to focus explicitly on the GRF could also be possible. Since a comparison of in‐flight training devices is needed that mimic as close as possible usual treadmill running on earth, the additional suspension system in upright treadmill running would be an additional influencing factor. Improving the effectiveness in applied external loading and receiving the closest GRF is still a big step to take (Schaffner et al., 2005). This is not only of concern for maladaptation in the musculoskeletal system, where forces and tissue strains play an important role (Frost, 1987). It also sheds new light on the old observation that the maintenance of the maximal cardiovascular performance level in Space poses a challenge and that refinement of the existing countermeasures is needed (Convertino, 1996).

An important outcome of our study is hypogravity simulation models have their specific imperfections and limitations. Given the strong dependence of ${\dot{V}O}_{2}$ on the treadmill reaction force, it must be suspected that astronauts perform their treadmill training at rather low metabolic rate at adaption phase which rises to the maximum achievable comfort loading of 0.7–0.8 g on a treadmill (Petersen et al., 2016), that are provided by bungee cords, which are known to decrease by 4–14% during running and walking (Schaffner et al., 2005). To give an example, according to the beta‐coefficients given under “Results,” a reduction in the GRF to 0.7 g will lower the ${\dot{V}O}_{2}$ by 20% when running at 10 km/h, and a reduction to 0.5 g will reduce ${\dot{V}O}_{2}$ by 33%. Such reductions, which are known to be characteristic for treadmill training on the International Space Station, must be expected to compromise the countermeasure effectiveness for cardiovascular crew health. Therefore, future countermeasure devices for deep space missions need to better address a feasible aerobic fitness component. On the other hand, it has recently been speculated that restricted energy ingestion in space would drive muscle wasting, and it has been suggested to banish aerobic exercises from astronaut countermeasure exercise regimens (Laurens et al., 2019). Results from the present study can be helpful to get a more realistic estimate of the true metabolic demands of treadmill running, for example, by including the actual treadmill forces on the International Space Station treadmill into computations of metabolic turnover.

## 5 CONCLUSION

The present study found significant differences in the cardiopulmonary response between supine running in a simulated micro gravitational environment and upright running on a regular treadmill. The reduction in ${\dot{V}O}_{\text{2max}}$ and HR_max_ throughout the supine conditions was well explained when considering the true GRFs, thus supporting the idea that g load and running speed define metabolic turnover.

In terms of exercising in Space, these results suggest that the impossibility of replacing full 1‐g loading in microgravity poses a limiting factor for aerobic exercise. This has implications for long‐term crew health in deep space missions.

## CONFLICT OF INTERESTS

The authors declare that there is no conflict of interest.

## AUTHORS CONTRIBUTIONS

The study was conducted in the Physiology laboratory of the Institute of Aerospace Medicine in the department of Space Physiology at the German Aerospace Center (DLR) in Cologne, Germany. All authors have contributed to‐conception or design of the work (KY,KM,JR, and AJ) or ‐ acquisition, analysis or interpretation of data for the work (KY,KM,MB,JB,AJ, and JR) and ‐ drafting the work or revising it critically for important intellectual content(KY, MB,JR,AJ,KM, and JB) KY, KM, AJ, and JR, designed the study and are responsible for the study conception. KY, KM, JB, and JR conducted the experiments and collected the data. KY, KM, MB, JB, AJ, and JR analyzed and interpreted the data. KY, MB, AJ, and JR drafted the work and KY, KM, MB, JR, AJ, and JB revised it critically for important intellectual content. All authors approved the final version of the manuscript and agree to be accountable for all aspects of the work in ensuring that questions related to the accuracy or integrity of any part of the work are appropriately investigated and resolved. All persons designated as authors qualify for authorship, and all those who qualify for authorship are listed.

## Supporting information



Supplementary Material

## Data Availability

The data that support the findings of this study are available in the supplementary material of this article.
