# Supplementary material for: Influence of simulated hypogravity on oxygen uptake during treadmill running
Source: Physiol Rep. 2021 May 5;9(9):e14787. doi: 10.14814/phy2.14787 (PMC8100405; doi:10.14814/phy2.14787)
Supplement: Supplementary file 1 — Supplementary Material [file PHY2-9-e14787-s001.pdf]

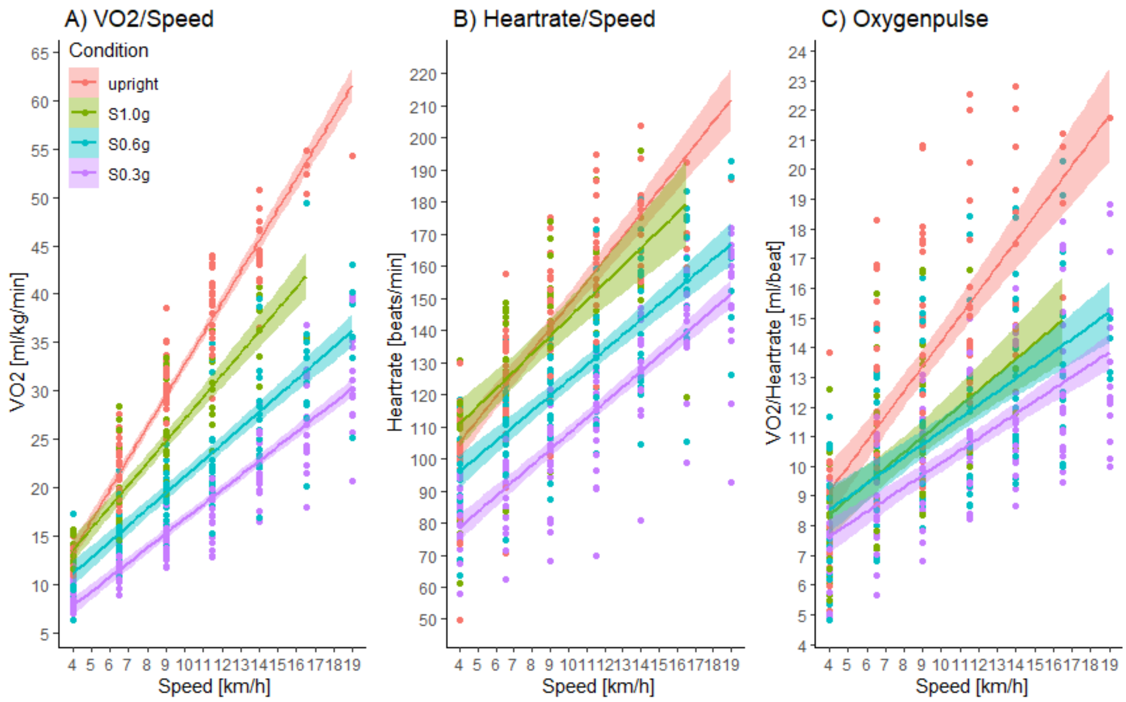

Supporting information Figure 4: Cardiorespiratory responses as a function of running speed, separately displayed for the 4 different gravitational conditions tested. The solid lines denote the regression lines of each condition whilst the shaded colors represent the standard errors with 95% of confidence interval.
Figure 3A: Maximum reached oxygen uptake in [ml /(kg*min)] in relation to running speed in [kilometre/h]
Figure 3B: Maximum reached heartrate in [beats/min] in relation to running speed in [kilometre/h]
Figure 3C: Oxygenpulse in [ml /heartbeat] as a function of running speed in [kilometre/h]

Visits	Protocol	Positon	G-Loading	Target
V0	Bruce	Upright<br>Supine	1.00<br>0.30-1.00	$\dot{V}O_2$ max<br>familiarization
V1	Incremental	Upright	1.00	Maximal Speed
V2-V4	Incremental	Supine	0.30, 0.60, 1.00 in randomized order	Maximal Speed

Supporting information Table 4: Overview of the five visits that were performed. “$\dot{V}O2_{max}$”: Maximum Oxygen uptake

Time in minutes	0-5	5-10	10-15	15-20	20-24	24-28	28-32	32-36	36-40	40-44	44-48	48-52	52
Speed in km/h	0	25% $\dot{V}O_{2max}$	50% $\dot{V}O_{2max}$	0	4	6.5	9	11.5	14	16.5	19	4 cool down	stop

Supporting information Table 5: Speed profile during visits V1-V4
